# Risk behavior for obesity: a conceptual analysis according to Walker and Avant

**DOI:** 10.1590/0034-7167-2024-0293

**Published:** 2025-07-11

**Authors:** Vinícius Rodrigues de Oliveira, Amanda Soares, Allyne Fortes Vitor, Maria Isabel da Conceição Dias Fernandes, Francisca Marta de Lima Costa Souza, Jonas Sâmi Albuquerque de Oliveira

**Affiliations:** I Universidade Federal do Rio Grande do Norte. Natal, Rio Grande do Norte, Brazil

**Keywords:** Obesity, Body Weight Maintenance, Risk, Risk-Taking, Concept Formation., Obesidad, Mantenimiento del Peso Corporal, Riesgo, Asunción de Riesgos, Formation de Concepts.

## Abstract

**Objective:**

To analyze the concept of risk behavior for obesity using the Walker and Avant method.

**Methods:**

A theoretical study employing conceptual analysis, operationalized through a scoping review. Searches were conducted in the following data sources: BVS, MEDLINE, Embase, and Scopus. Studies available in full text were included, while duplicates and those not aligned with the review’s theme were excluded.

**Results:**

The conceptual analysis was based on 16 articles, which highlighted the most prevalent use of the concept in the fields of public health and nutrition. Twelve defining attributes related to diet, physical activity, and general habits were identified. Among the antecedents and consequences, the most notable were gender and age group as antecedents, and increased susceptibility to overweight/obesity as a consequence.

**Final Considerations:**

The analysis contributed to clarifying the concept of risk behavior for obesity. It is recommended to apply this concept in Nursing practice.

## RESUMEN

**Objetivo:**

Analizar el concepto de comportamiento de riesgo para la obesidad utilizando el método de Walker y Avant.

**Métodos:**

Estudio teórico, del tipo análisis conceptual, operacionalizado a través de una revisión de alcance (*scoping review*). Las búsquedas se realizaron en las siguientes fuentes de datos: BVS, MEDLINE, Embase y Scopus. Se incluyeron estudios disponibles en su totalidad, excluyéndose aquellos duplicados o que no se ajustaban al tema de la revisión.

**Resultados:**

El análisis conceptual se basó en 16 artículos que señalaron el uso más prevalente del concepto en el campo de la salud pública y la nutrición. Se identificaron 12 atributos definitorios relacionados con la alimentación, la actividad física y los hábitos generales. Entre los antecedentes y los consecuentes, destacaron, respectivamente, la influencia del género y del grupo etario, y la mayor susceptibilidad al sobrepeso/obesidad.

**Consideraciones Finales:**

El análisis contribuyó a la definición del concepto de comportamiento de riesgo para la obesidad. Se recomienda la aplicación de este concepto en la práctica de Enfermería.

## INTRODUCTION

Currently, the global epidemiological landscape is marked by an increase in Non-Communicable Diseases (NCDs), among which obesity stands out. This condition has shown rapid progression and is difficult to control, resulting in its classification as a pandemic^(1)^. Estimates indicate that, in 2022, approximately one billion people of various age groups were living with obesity^(2)^.

Obesity has negatively impacted global health and well-being indicators, establishing itself as one of the greatest public health challenges of the 21st century. The progressive rise in obesity rates is associated with numerous health complications, including cardiovascular, endocrine, and metabolic diseases, as well as psychological disorders. Additionally, the costs related to managing obesity, excluding associated pathologies, exceed millions of dollars annually and are expected to increase proportionally with the progression of the disease^(3)^.

Recognized as a complex and multifactorial disease, obesity results from the interaction of genetic, environmental, and behavioral factors^(4)^. In this context, research has emphasized the need to investigate risk behaviors as a strategy to identify more effective ways to manage the disease^(5,6)^.

Risk behavior is defined as actions that expose individuals to threats of death or harm to their health, whether immediate or long-term^(7)^. When associated with a specific illness context, this concept acquires new meanings, as in the case of risk behavior for obesity. This term, derived from the general concept of risk behavior, often lacks a clear definition or is implicitly understood in some circumstances.

Thus, it is crucial to conduct studies that elucidate the concept of risk behavior for obesity, enabling the term to be appropriately integrated into the care of individuals at risk of developing the disease without inappropriate associations. According to the guidelines of the Brazilian Ministry of Health, such care should primarily take place in Primary Health Care (PHC), involving a multidisciplinary team, including nurses who play a vital role in implementing promotion and prevention actions to combat obesity^(8)^.

Clarifying this concept can contribute to the development of more targeted interventions and the formulation of public health policies focusing on the most relevant and prevalent behaviors associated with obesity. A precise definition of the concept of risk behavior for obesity also facilitates the reduction of such behaviors in the population.

To explore this term, a concept analysis is recommended, defined as a “rigorous process to bring clarity to the definition of concepts used in science”^(9)^. This method, widely utilized in nursing, supports both research and practice in the field.

## OBJECTIVE

To analyze the concept of risk behavior for obesity according to the Walker and Avant method.

## METHODS

### Ethical aspects

This study used only data in the public domain and did not involve human participants. Therefore, there was no requirement for review by a Research Ethics Committee.

### Study design

This is a theoretical study based on the conceptual analysis method developed by Walker and Avant. The method is operationalized through eight stages: selecting the concept to be analyzed, defining the objectives of the analysis, determining the possible uses of the concept, identifying the defining attributes, presenting a model case, presenting other types of cases, identifying antecedents and consequences related to the concept, and, finally, designating empirical referents^(10)^. It is important to note that this study covered the first seven stages, which were considered sufficient to achieve the proposed objective.

The term selected for analysis was “risk behavior for obesity.” This choice was based on the author’s familiarity with the topic of obesity and the need to clarify the concept to advance knowledge and support future studies in this field. The following objectives were defined for the analysis:

- Develop a definition of risk behaviors for obesity;
- Determine possible uses, defining attributes, antecedents, and consequences related to risk behavior for obesity.

It is worth noting that antecedents are events that precede the concept and are considered precursors to its occurrence, whereas consequences are events that result from the presence of the concept’s attributes^(11)^.

To conduct the stages of the analysis, a scoping review of the literature was performed, following the guidelines of the *JBI Manual for Evidence Synthesis* ^(12)^. The protocol was registered on the Open Science Framework (OSF) platform and is available at https://osf.io/657j3/.

Initially, the platforms *International Prospective Register of Systematic Reviews* (PROSPERO), Open Science Framework (OSF), and *The Cochrane Library* were accessed to identify studies with objectives similar to those of the present review. However, no analogous studies were identified.

### Inclusion and Exclusion Criteria

The eligibility criteria included studies available in full text, free of charge, in any language or year of publication. Duplicated studies or those outside the thematic scope of the review were excluded.

### Study Protocol and Period

Based on the PCC strategy (Population, Concept, and Context), the following research questions were established: “What is the concept of risk behavior for obesity? What are its uses, defining attributes, antecedents, and consequences?”

The selection of studies for the development of this conceptual analysis was carried out through access to the *Portal de Periódicos* of the *Coordenação de Aperfeiçoamento de Pessoal de Nível Superior* (CAPES). The following data sources were consulted: *Biblioteca Virtual em Saúde* (BVS), Medical Literature Analysis and Retrieval System Online (MEDLINE), Embase, and Scopus. The search was conducted concurrently by two researchers with nursing backgrounds and expertise in obesity, both affiliated with the Universidade Federal do Rio Grande do Norte (UFRN).

To construct the search strategy, *Descritores em Ciências da Saúde* (DeCS) were used for platforms in Portuguese, and Medical Subject Headings (MeSH) for English-language databases. For Embase, Emtree terms were utilized instead. The descriptors were combined using the Boolean operators AND and OR.

The search strategies, tailored to each data source, were as follows:

- Search 01 - conducted in BVS: (“*obesidade*”) OR (“*Manutenção do Peso Corporal*”) AND (“*Comportamento de risco*”);
- Search 02 - conducted in BVS: (*obesidade*) AND (*comportamento obesogênico*);
- Search 03 - conducted in Embase: ((obesity) OR (Body Weight Maintenance)) AND (‘high risk behavior’);
- Search 04 - conducted in Embase: ((obesity) AND (“obesogenic behaviors”));
- Search 05 - conducted in MEDLINE and Scopus: (obesity) OR (Body Weight Maintenance) AND (Risk-Taking);
- Search 06 - conducted in MEDLINE and Scopus: ((obesity) AND (“obesogenic behaviors”)).

After conducting the searches, the results obtained by each researcher were reviewed and verified. The selected publications were exported to the software Rayyan (version 2016, Qatar Foundation), where an initial screening was performed based on the reading of titles and abstracts. The studies preliminarily selected were then read in full to confirm their inclusion in the final sample.

The selection process was conducted by a nurse researcher specializing in obesity, ensuring the relevance and quality of the studies included.

### Analysis of Results

The study data were analyzed using Laurence Bardin’s Content Analysis (CA) methodology. The operationalization of CA consists of three phases: pre-analysis, material exploration, and result interpretation^(13)^. Briefly, the application of this technique to the study context unfolded as follows:

In the first phase, the researcher conducted a preliminary reading of the studies in the sample, extracting initial impressions of the material and mentally organizing the accessed information. In the second phase, the researcher delved deeper into the material, developing specific processes such as coding and categorization of the information. Finally, in the third phase, the researcher employed inference techniques to interpret the content, attributing meaning to the study data to construct the concept.

## RESULTS

**Figure 1:**
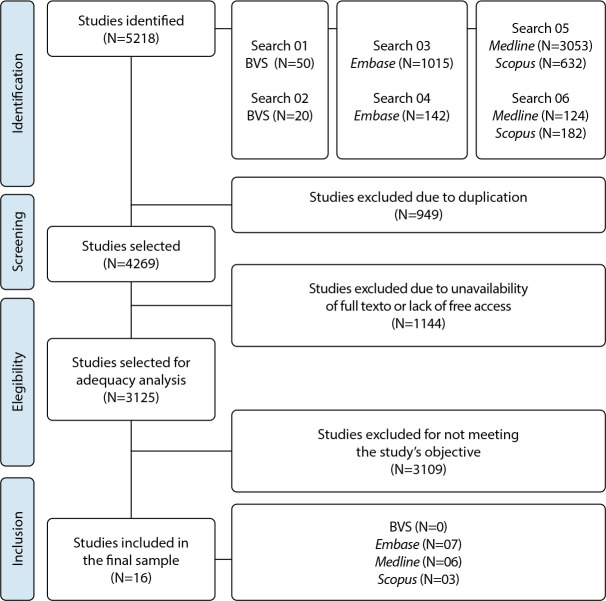
Flowchart of study selection for sample composition, Brazil, 2025

A total of 5,218 studies were identified in the initial search, conducted using only the descriptors and Boolean operators. This material was exported to Rayyan software, where a screening process was performed. Inclusion and exclusion criteria were applied, resulting in a final sample of 16 studies. The selection process is detailed in Figure 1.

The 16 publications included in the final sample primarily consisted of quantitative cross-sectional studies (50%) conducted in nine different countries, with the United States (37.5%) and Brazil (18.7%) standing out. The studies were published over a 15-year period (2008-2023), with the highest prevalence in 2017 (25%). The target populations included adolescents (75%), children (12.5%), adults (6.25%), and workers (6.25%). Regarding the language of publication, the studies were available in English (93.7%) and Portuguese (6.3%).

In the conceptual analysis, it was found that the term *risk behavior for obesity* was most frequently used in the fields of Public Health (31.25%) and Nutrition (25%). Additionally, alternative terminologies were identified, such as *behavioral risk factors for overweight* ^(14)^, *obesogenic behavior* ^(15-17)^, and *risk behavior for overweigh^t^* ^(18)^. Notably, none of the studies provided a formal definition for *risk behavior for obesity* or any equivalent term.

Twelve attributes were identified, grouped into three main categories: diet, physical activity, and general habits. The central attributes in each category were as follows: consumption of sugary beverages associated with diets high in carbohydrates and fat (56.25%); physical inactivity (75%); and excessive screen time (56.25%). Chart 1 provides detailed information about the results.

**Chart 1:** Uses and attributes of the concept of risk behavior for obesity, Brazil, 2025

Uses of the concept
Public Health^(14,15,18-20)^
Nutrition^(2-24)^
Medicine^(17,25,26)^
Physical Education^(27,28)^
Nursing^(16,29)^
**Defining attributes of the concept**
**Related to diet:** -Consumption of sugary beverages^(14,16,17,20-24,28)^ -Diets high in carbohydrates and fat^(14,19-25,28)^ -Low intake of fruits and/or vegetables^(16,17,20-26)^ -Skipping breakfast^(14-16,18,20,24,25)^ -Frequent consumption of fast food and ultra-processed foods^(15,16,19-21,29)^
**Related to physical activity:** -Physical inactivity^(16-26,28)^ -Sedentary behavior^(20,21,25,28)^ -Lack of participation in physical education classes^(24)^
**Related to general habits:** -Excessive screen time^(14,15,17-21,26,29)^ -Smoking^(16,20,21,26,27)^ -Alcohol consumption^(19-21,28,29)^ -Eating while watching television^(15,24,29)^

After selecting the attributes, the model case and the related case were constructed (Chart 2). Developing these cases is essential for enhancing the reader’s understanding of the concept, as the model case demonstrates a practical example of its application. In contrast, the related case, while presenting attributes similar to the concept, also includes elements that do not align with its scope when analyzed in depth^(10)^.

**Chart 2:** Model case and borderline case related to the concept of risk behavior for obesity, Brazil, 2025

Model Case
Nurse Anna, while collecting anthropometric data from schoolchildren, attended to A.S.M., a 16-year-old Black adolescent weighing 74.7 kg, with a height of 162 cm and a Z-score BMI of +2, corresponding to a classification of obesity. After identifying the condition, the nurse initiated a conversation with the adolescent to discuss his health situation. During the initial approach, she completed a food consumption form and discussed lifestyle habits to identify risk behaviors for obesity and plan an intervention based on the findings.During the conversation, the adolescent reported that he did not consume fruits or vegetables, except for potatoes, which he mentioned liking, particularly “French fries with cheddar and bacon.” Additionally, he stated that he preferred soft drinks over fruit juices. A.S.M. also revealed that he skipped breakfast in the mornings because he often woke up late for school due to poor sleep quality at night. Throughout the day, he reported consuming quick snacks, such as stuffed cookies or fried savory snacks. For dinner, he mentioned frequently eating instant noodles because it was a more practical option, as his mother worked outside the home, leaving him responsible for preparing his meals. Finally, the adolescent stated that he did not engage in physical activities, not even during school physical education classes, and instead preferred watching series or playing video games.
**Related Case**
V.C.O., a 17-year-old female, visited the Basic Health Unit for a family planning consultation. The patient has Polycystic Ovary Syndrome (PCOS). Before the consultation, her anthropometric measurements were taken, revealing a Z-score BMI of +3, indicative of obesity. The nurse conducting the consultation began by addressing the patient’s nutritional status. V.C.O. reported being aware of her condition and believed that her situation was related to genetic factors, as obesity cases were frequent on her maternal side of the family. Additionally, she stated that her excess weight might be associated with PCOS and/or the use of antidepressants. The patient also disclosed that she did not engage in physical activity and spent a significant amount of time using her phone. In response, the nurse provided brief recommendations regarding physical activity and reducing excessive screen time. The consultation then continued, focusing on the patient’s family history and PCOS while emphasizing concerns related to the diagnosis of obesity.

**Figure 2:**
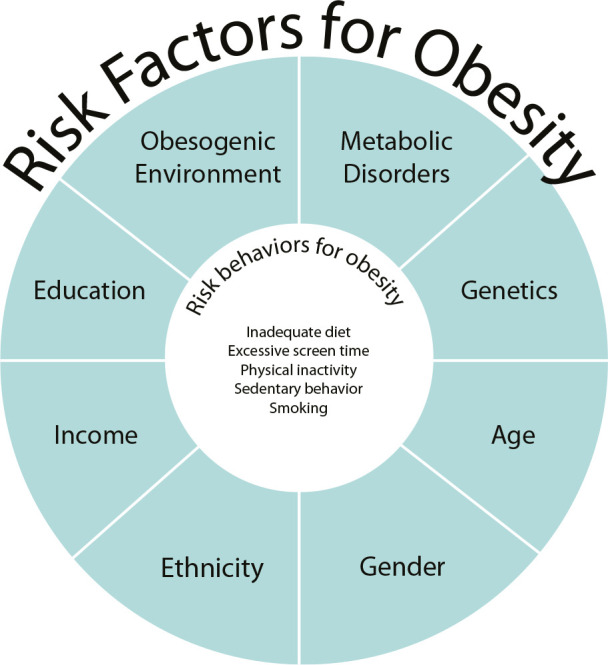
System of risk factors for obesity and its association with risk behaviors for obesity, Brazil, 2024

It is noteworthy that among the possible complementary cases, the related type was chosen because the reviewed literature revealed conceptual inconsistencies between risk factors and risk behaviors. While the related case includes some elements typical of risk behavior for obesity, the majority pertain to risk factors, such as age, family history, pre-existing conditions, and the use of certain medications. To further clarify this distinction, Figure 2 was developed, illustrating the association between risk behaviors and risk factors for obesity.

Continuing with the steps of the concept analysis, 11 antecedents and six consequences were identified. The primary antecedents included the influence of gender and age group (68.75%) and unfavorable socioeconomic conditions (43.75%). Among the consequences, increased susceptibility to overweight/obesity (100%) and a heightened risk of developing chronic endocrine and cardiovascular diseases, as well as cancers (68.75%), stood out. Chart 3 below presents the findings in detail.

**Chart 3:** Antecedents and consequences of the concept of risk behavior for obesity, Brazil, 2024

Antecedents	Consequences
-Influence of gender and age group^(16-18,20,22,24,27-29)^ -Unfavorable socioeconomic conditions^(16-21,23,25,26)^ -Food insecurity^(17,19,21,23,26)^ -Obesogenic home or school environment^(15,16,19,22,23)^ -Sexual, racial, or ethnic minority groups^(14,17,26,29)^ -Lack of parental control/monitoring^*(14,15,29)^ -Absence of support/encouragement to avoid the behavior^(19-21)^ -Need for social approval^(16,19,27)^ -Influence of social media and advertisements for obesogenic foods^(15,19,24)^ -Unfavorable environment for physical exercise^**(17,19,21)^ - Limited nutritional knowledge among caregivers^*(23)^	-Greater susceptibility to overweight and obesity^(14-29)^ -Increased risk of developing chronic endocrine and cardiovascular diseases and cancers^(15,17,19-22,24,25,28,29)^ -Physical and mental health impairments in later life stages^(14-16,24,29)^ -Reduced sleep duration and quality^(15,16,18)^ -Premature death^(25,28)^ - Triggering of other risk behaviors ^(20,24)^

Legend: Specific to children and adolescents

** Considerations include safety issues and limited physical spaces.

## DISCUSSION

As observed in the results, studies on risk behavior for obesity have been conducted worldwide, reflecting the global trend of increasing excess weight, particularly in developing countries such as Brazil^(30)^. However, the United States, the country with the highest representation in the sample, despite not being classified as a developing nation, also exhibits high obesity rates^(31)^.

Given the worsening of this condition and its repercussions on individuals’ lives and healthcare systems, research on the subject has become increasingly necessary. However, as identified in the present sample, there is a predominance of quantitative studies, which limits a more comprehensive exploration of the subjective aspects of illness, such as behavior^(32)^.

Scientific evidence indicates that individual behaviors are directly associated with obesity. In this context, it is clear that investigating risk behaviors is essential for addressing obesity^(14-29)^. Despite its importance, the topic remains underexplored from a conceptual perspective and is often confused with other concepts.

Even in the fields of public health and nutrition, where the term has been most frequently addressed, a solid definition is lacking. The studies analyzed tend to cite examples of risk behaviors for obesity but fail to provide a precise definition. The same issue arises in fields such as medicine, nursing, and physical education, where this terminology or its synonyms are also identified.

The use of the term in public health underscores the recognition of multiple factors influencing obesity. In this context, discussions often include the development of public policies aimed at addressing situations that increase the risk of obesity, contrasting, for example, with the actions of companies that, through their products or practices, promote unhealthy behaviors^(33)^.

In the health sciences (nutrition, medicine, nursing, and physical education), the importance of multiprofessional care for individuals with obesity or at risk of developing it is emphasized. Recommendations from the Ministry of Health suggest that professionals, particularly those working in PHC, such as doctors and nurses, should be trained to intervene in obesity and identify potential risks contributing to the disease, including behaviors. This responsibility should not fall exclusively on nutritionists. Additionally, involving physical education professionals and psychologists is a strategic approach to combating obesity^(8)^.

From an analytical perspective of the concept, the defining attributes of risk behavior for obesity are centered on three main dimensions: diet, physical activity, and general habits. The correlation of these attributes with obesity was expected and is extensively documented in the scientific literature.

The studies included in the sample make a significant contribution by highlighting the need to investigate these behaviors in an integrated manner rather than addressing them separately. Considering that obesity is a multifactorial condition, the importance of analyzing behaviors and risk factors together is reinforced, ensuring that interventions are more effective^(16,20,21,24,28-29)^.

The defining attributes related to diet refer to unhealthy eating practices characterized by skipping certain meals throughout the day and consuming foods with low nutritional value, high energy content, and elevated levels of sugars and preservatives. These practices generally vary among adults, adolescents, and children.

For adults, risk behaviors related to diet include issues such as the high cost of healthy foods, a lack of time to prepare meals, the need to maintain motivation to adhere to a balanced diet, and limited nutritional knowledge^(34)^. In contrast, the eating habits of adolescents and children are influenced by social factors within family and school environments, such as peer acceptance and parental control. The latter is particularly relevant among children, as their food choices depend on their caregivers or guardians^(15,16,19,22,23)^.

Regarding attributes linked to physical activity, the primary factors are sedentary behavior and physical inactivity. Sedentary behavior is associated with low energy expenditure due to activities such as remaining seated or lying down for long periods, whereas physical inactivity refers to the absence of exercise^(35)^. These behaviors can be addressed through various approaches, with motivational strategies standing out as particularly effective^(36)^.

Among adolescents, a specific attribute identified was the lack of participation in practical physical education classes. Studies indicate that failing to engage in these classes is detrimental, as individuals who participate in school physical education tend to achieve higher levels of physical activity throughout the week compared to those who do not attend the classes^(37)^.

Finally, the third category of attributes pertains to general habits associated with risk behavior for obesity, including excessive screen time, smoking, and alcohol consumption. While the literature widely emphasizes dietary and sedentary behaviors, screen use and substance consumption, such as alcohol and tobacco, should also be considered fundamental in addressing patients with obesity or those at risk of developing it^(20)^.

Among these factors, excessive screen time warrants particular attention, as it triggers other risk behaviors. This habit is recognized as a marker of sedentary behavior, is more prevalent among children and adolescents, and is associated with the development of obesity and overweight^(17,29)^.

Concerning the antecedents of the concept, these were mostly associated with demographic, nutritional, and socioenvironmental aspects. Notable factors included gender, age, ethnicity, socioeconomic conditions, food insecurity, and obesogenic environments. Other antecedents, not fitting into these categories, were less frequently identified in the analyzed sample.

Although there was no consensus among studies regarding the influence of gender on risk behavior, a significant portion indicated that males are more likely to develop such behaviors^(16,20-22)^. Similarly, adolescents were identified as the age group most susceptible to adopting risk behaviors in general, many of which are considered predictors of obesity^(15-21,26,27,29)^.

Regarding food insecurity, this condition arises when the principles of food security are violated-that is, when adequate access, quality, and quantity of food are not guaranteed. It is noteworthy that there is a close relationship between low socioeconomic conditions and food insecurity, both of which are significant risk factors for the development of obesity^(38)^.

With respect to obesogenic environments, these can be defined as spaces where individuals are negatively influenced to adopt unhealthy dietary habits and sedentary behavior patterns, leading to adverse health outcomes^(39)^. There is no single location that can be exclusively classified as obesogenic, but any environment fitting this definition can be categorized as such.

In the school context, studies indicate that factors such as the availability of ultra-processed foods and fast food within or near schools, inadequate infrastructure (marked by a lack of spaces for physical activities), and insecurity in the surrounding area are the primary contributors to students’ adoption of risk behaviors for obesity^(40)^.

Additionally, peer influence and the need for social acceptance often lead adolescents to adopt unhealthy eating practices and other risk behaviors as a means of gaining validation^(41)^. Compounding this is the role of the media, which promotes advertisements and content that normalize and encourage unhealthy dietary habits, making them appear commonplace^(42)^.

In light of this scenario, parents play a crucial role in promoting healthy habits. They can encourage adolescents and children to develop positive practices, such as maintaining a balanced diet, engaging in regular physical activity, and monitoring screen time. Furthermore, parents should serve as positive role models by adopting healthy behaviors in their own lives^(43)^.

Concerning the consequences of the concept, it is essential to emphasize that identifying them provides a broad understanding of the complications and harms associated with obesity, which are strongly influenced by risk behaviors. These findings also underscore the propensity of individuals who engage in risk behaviors to develop the disease and experience associated physical and psychological harms, leading to a reduced quality of life that can persist into adulthood without intervention.

Given this situation, a study^(44)^ highlights the importance of implementing interventions as early as possible to prevent complications from developing. Primary prevention, particularly through educational interventions, is an effective approach to achieving positive outcomes in addressing the issue, as demonstrated by research^(45)^.

### Study Limitations

The primary limitation of this study was the exclusive use of electronic sources for the search, combined with the lack of consultation with gray literature and the exclusion of many studies due to restricted free access, even though the searches were conducted through the CAPES Journal Portal. Additionally, the difficulty in identifying and associating descriptors that adequately reflected the subject in question is noteworthy.

### Contributions to the Fields of Nursing, Health, or Public Policy

The conceptual analysis of the term *risk behavior for obesity* provides theoretical support to foster critical thinking, evidence-based practice, and diagnostic reasoning in nursing care. Furthermore, it enhances the understanding of the underlying factors of obesity, enabling nurses to act as health promoters and facilitators of a healthy lifestyle in any service within the Health Care Network.

## FINAL CONSIDERATION

This analysis, conducted using the Walker and Avant method, clarified the concept of *risk behavior for obesity,* which had previously remained undefined or, at best, implied. Based on the literature reviewed, the authors define *risk behavior for obesity* as an action undertaken by an individual, most frequently during adolescence (though not exclusively during this stage), that increases susceptibility to obesity and its complications, including chronic cardiometabolic diseases and a higher risk of premature death.

In defining this term, its antecedents, defining attributes, and consequences were identified, allowing for a deeper understanding and broader knowledge by the authors. This facilitated breaking away from inappropriate associations that did not align with the concept.

Moreover, it is suggested that future research validate *risk behavior for obesity* as a nursing diagnosis, considering that taxonomies providing theoretical and clinical support for nursing practice, such as the North American Nursing Diagnosis Association (NANDA), already include a diagnosis related to obesity.

## References

[1] Popkin BM, Adair LS, Ng SW. (2012). Global nutrition transition and the pandemic of obesity in developing countries. Nutr Rev.

[2] World Health Organization (WHO) (2021). Overweight and obesity.

[3] Alfaris N, Alqahtani AM, Alamuddin N, Rigas G. (2023). Global Impact of Obesity. Gastroenterol Clin North Am.

[4] Batterham RL. (2020). Switching the focus from weight to health: Canada’s adult obesity practice guideline set a new standard for obesity management. EClinicalMedicine.

[5] Wilfley DE, Hayes JF, Balantekin KN, Van Buren DJ, Epstein LH. (2018). Behavioral interventions for obesity in children and adults: evidence base, novel approaches, and translation into practice. Am Psychol.

[6] Pelletier JE, Lytle LA, Laska MN. (2016). Stress, health risk behaviors, and weight status among community college students. Health Educ Behav.

[7] Zappe JG, Alves CF, Dell’aglio DD. (2018). Comportamentos de risco na adolescência: revisão sistemática de estudos empíricos. Psicol Rev.

[8] Ministério da Saúde (BR) (2022). Manual de atenção às pessoas com sobrepeso e obesidade no âmbito da Atenção Primária à Saúde (APS) do Sistema Único de Saúde. Ministério da Saúde.

[9] McEwen M, Wills EM. (2016). Bases teóricas de enfermagem.

[10] Walker L, Avant KC., Walker L, Avant KC. (2011). Strategies for theory construction in nursing.

[11] Fernandes M das GM, Nóbrega MML da, Garcia TR, Macêdo-Costa KN de (2011). F. Análise conceitual: considerações metodológicas. Rev Bras Enferm.

[12] Peters MDJ, Godfrey C, McInerney P, Baldini Soares C, Khalil H, Parker D., Aromataris E, Munn Z (2020). Joanna Briggs Institute Reviewer’s Manual.

[13] Bardin L. (2016). Análise de conteúdo.

[14] Veldhuis L, Vogel I, Renders CM, van Rossem L, Oenema A, HiraSing RA (2012). Behavioral risk factors for overweight in early childhood; the ‘Be active, eat right’ study. Int J Behav Nutr Phys Act.

[15] Mihrshahi S, Drayton BA, Bauman AE, Hardy LL. (2017). Associations between childhood overweight, obesity, abdominal obesity and obesogenic behaviors and practices in Australian homes. BMC Public Health.

[16] Lee H, La IS. (2021). Latent class analysis of obesogenic behaviors among Korean Adolescents: associations with Weight-Related Outcomes. Int J Environ Res Public Health.

[17] Rodriguez LA, Gopalan A, Darbinian JA, Chandra M, Greenspan LC, Howell A (2022). Identifying modifiable obesogenic behaviors among Latino adolescents in primary pediatric care. Prev Med Rep.

[18] Continente X, Pérez A, Espelt A, Ariza C, López MJ. (2017). Multiple lifestyle risk behaviours and excess weight among adolescents in Barcelona, Spain. Gac Sanit.

[19] Hendricks G, Savona N, Aguiar A, Alaba O, Booley S, Malczyk S (2022). Adolescents’ perspectives on the drivers of obesity using a group model building approach: a South African perspective. Int J Environ Res Public Health.

[20] Laxer RE, Brownson RC, Dubin JA (2017). Clustering of risk-related modifiable behaviours and their association with overweight and obesity among a large sample of youth in the COMPASS study. BMC Public Health.

[21] Boone-Heinonen J, Gordon-Larsen P, Adair LS. (2008). Obesogenic Clusters: Multidimensional Adolescent Obesity-related Behaviors in the U.S. Ann Behav Med.

[22] Cook AS, O’Leary F, Chey T, Bauman A, Allman-Farinelli M. (2013). Prevalence of and intention to change dietary and physical activity health risk behaviours. Appetite.

[23] Song WO, Song S, Nieves V, Gonzalez A, Crockett ET. (2016). Nutritional health attitudes and behaviors and their associations with the risk of overweight/obesity among child care providers in Michigan Migrant and Seasonal Head Start centers. BMC Public Health.

[24] Ferreira NL, Claro RM, Mingoti SA, Lopes ACS. (2017). Coexistence of risk behaviors for being overweight among Brazilian adolescents. Prev Med.

[25] Sanchez A, Norman GJ, Sallis JF, Calfas KJ, Rock C, Patrick K. (2008). Patterns and correlates of multiple risk behaviors in overweight women. Prev Med.

[26] Fournier ME, Austin SB, Samples CL, Goodenow CS, Wylie SA, Corliss HL. (2009). A comparison of weight-related behaviors among high school students who are homeless and non-homeless. J Sch Health.

[27] Silva DR, Ohara D, Tomeleri CM, Batista MB, Fernandes RA, Ronque ER (2016). Association between risk behaviors and adiposity indicators in adolescents from Southern Brazil: a methodological approach. J Child Health Care.

[28] Streb AR, Del Duca GF, Silva RP, Benedet J, Malta DC. (2020). Simultaneidade de comportamentos de risco para a obesidade em adultos das capitais do Brasil. Ciênc Saúde Coletiva.

[29] Silva TPR, Matozinhos FP, Gratão LHA, Rocha LL, Inácio MLC, Oliveira CF (2022). The coexistence of obesogenic behaviors among Brazilian adolescents and their associated factors. BMC Public Health.

[30] Chooi YC, Ding C, Magkos F. (2019). The epidemiology of obesity. Metabolism.

[31] Hales CM, Carroll MD, Fryar CD, Ogden CL. (2020). Prevalence of obesity and severe obesity among adults: United States, 2017-2018. NCHS Data Brief.

[32] Mariz LS, Enders BC, Santos VEP, Tourinho FSV, Vieira CENK (2015). Causes of infantile-juvenile obesity: reflexions based on the theory of Hannah Arendt. Texto Contexto Enferm.

[33] Martins APB (2018). É preciso tratar a obesidade como um problema de saúde pública. Rev Adm Empres.

[34] Lindemann IL, Oliveira RR, Mendoza-Sassi RA. (2016). Dificuldades para alimentação saudável entre usuários da atenção básica em saúde e fatores associados. Ciênc Saúde Coletiva.

[35] Franco FSC, Miranda TS, Lopes SC, Matos YA, Santos MEJ. (2022). Nível de atividade física e sedentarismo associados a imagem corporal em adolescentes antes e durante a pandemia do covid-19. Holos.

[36] Nogg KA, Vaughn AA, Levy SS, Blashill AJ. (2021). Motivation for Physical Activity among U.S. Adolescents: a self-determination theory perspective. Ann Behav Med.

[37] Zhan X, Clark CCT, Bao R, Duncan M, Hong JT, Chen ST. (2021). Association between physical education classes and physical activity among 187,386 adolescents aged 13-17 years from 50 lowand middle-income countries. J Pediatr.

[38] Araújo ML, Nascimento DR, Lopes MS, Passos CM, Lopes ACS. (2020). Condições de vida de famílias brasileiras: estimativa da insegurança alimentar. Rev Bras Estud Popul.

[39] Ministério da Saúde (BR) (2022). Ambiente obesogênico: você sabe o que é?.

[40] Filgueiras MS, Pessoa MC, Bressan J, Fogal Vegi AS, Carmo AS, Albuquerque FM (2023). Characteristics of the obesogenic environment around schools are associated with body fat and low-grade inflammation in Brazilian children. Public Health Nutr.

[41] Rodrigues EF, Gomes GC, Lorenção LG, Alvarez SQ, Pintanel AC, Ribeiro JP. (2020). The influence of friendships on adolescent’s behavior and health. Res Soc Dev.

[42] Bittar C, Soares A. (2020). Mídia e comportamento alimentar na adolescência. Cad Bras Ter Ocup.

[43] Piasetzki TRC, Boff ETO, Battisti IDE. (2020). Influência da família na formação dos hábitos alimentares e estilos de vida na infância. Rev Contexto Saúde.

[44] Lee EY, Yoon KH. (2018). Epidemic obesity in children and adolescents: risk factors and prevention. Front Med.

[45] Gato-Moreno M, Martos-Lirio MF, Leiva-Gea I, Bernal-López MR, Vegas-Toro F (2021). Early nutritional education in the prevention of childhood obesity. Int J Environ Res Public Health.

