# Supplementary material for: Risk behavior for obesity: a conceptual analysis according to Walker and Avant
Source: Rev Bras Enferm. 2025 Jul 11;78(3):e20240293. doi: 10.1590/0034-7167-2024-0293 (PMC12252927; doi:10.1590/0034-7167-2024-0293)
Supplement: Translation

# Translation: Comportamento de risco para obesidade: análise conceitual de acordo com Walker e Avant

**DOI:** 10.1590/0034-7167-2024-0293pt

**Authors:** Vinícius Rodrigues de Oliveira, Amanda Soares, Allyne Fortes Vitor, Maria Isabel da Conceição Dias Fernandes, Francisca Marta de Lima Costa Souza, Jonas Sâmi Albuquerque de Oliveira

**Affiliations:** I Universidade Federal do Rio Grande do Norte. Natal, Rio Grande do Norte, Brasil

**Keywords:** Obesidade, Manutenção do Peso Corporal, Risco, Comportamento de Risco, Formação de Conceito.

## Abstract

**Objetivo:**

Analisar o conceito de comportamento de risco para obesidade utilizando o método de Walker e Avant.

**Métodos:**

Estudo teórico, do tipo análise de conceito, operacionalizado por meio de uma *scoping review*. As buscas foram realizadas nas seguintes fontes de dados: BVS, MEDLINE, Embase e Scopus. Foram incluídos estudos disponíveis na íntegra, sendo excluídos aqueles duplicados ou que não se adequavam ao tema da revisão.

**Resultados:**

A análise conceitual baseou-se em 16 artigos, que apontaram o uso mais prevalente do conceito no campo da saúde pública e da nutrição. Foram identificados 12 atributos definidores relacionados à alimentação, atividade física e hábitos gerais. Dentre os antecedentes e consequentes, destacaram-se, respectivamente, a influência do gênero e da faixa etária, e a maior suscetibilidade ao sobrepeso/obesidade.

**Considerações finais:**

A análise contribuiu para a definição do conceito de comportamento de risco para obesidade. Recomenda-se a aplicação desse conceito na prática de Enfermagem.

## INTRODUÇÃO

Na atualidade, o cenário epidemiológico mundial é marcado pelo aumento das Doenças Crônicas Não Transmissíveis (DCNTs), entre as quais se destaca a obesidade. Essa doença tem demonstrado rápida evolução e difícil controle, o que favoreceu a aquisição do status de pandemia^(1)^. Estimativas revelam que o número de pessoas com obesidade, em 2022, era de aproximadamente um bilhão de indivíduos, abrangendo diferentes faixas etárias^(2)^.

A obesidade tem desestabilizado os indicadores globais de saúde e bem-estar, tornando-se um dos maiores desafios de saúde pública do século XXI. O aumento progressivo nas taxas da doença está associado a diversas complicações de saúde, como o surgimento de doenças cardiovasculares, endócrinas e metabólicas, além de transtornos psicológicos. Acrescem-se ainda os gastos com seu manejo que, sem considerar as patologias colaterais, giram em torno de milhões de dólares ao ano e tendem a acompanhar seu ritmo de crescimento^(3)^.

Considerada uma doença complexa e multifatorial, a obesidade resulta de um conjunto de fatores genéticos, ambientais e comportamentais^(4)^. Nesse contexto, estudos indicam a necessidade de investigar o comportamento de risco como meio de identificar estratégias adequadas para o manejo da doença^(5,6)^.

O comportamento de risco é definido como atitudes que colocam o indivíduo sob ameaça de morte ou exposto a danos precoces ou tardios^(7)^. Esse conceito, quando associado a um contexto de adoecimento específico, ganha um novo significado, como, por exemplo, o comportamento de risco para obesidade. O referido termo, originado a partir da adaptação do conceito de comportamento de risco para o cenário do adoecimento por obesidade, não está claramente definido ou, por vezes, é subentendido.

Nesse sentido, é importante realizar estudos que elucidem o conceito de comportamento de risco para obesidade, de modo que o termo possa ser devidamente implementado na assistência às pessoas em risco de obesidade, evitando associações inadequadas. Essa assistência, conforme recomenda o Ministério da Saúde do Brasil, deve ocorrer, essencialmente, na Atenção Primária à Saúde (APS), com a participação de uma equipe multiprofissional, da qual o enfermeiro faz parte, executando ações promotoras e preventivas no combate à obesidade^(8)^.

Ressalta-se que, ao clarificar esse conceito, os autores contribuirão para o desenvolvimento de intervenções assertivas e para o direcionamento de políticas públicas de saúde, cujo enfoque esteja nos comportamentos mais relevantes e prevalentes em relação à obesidade. Sobretudo, uma definição precisa sobre o conceito de comportamento de risco para obesidade tende a reduzir esses comportamentos.

Para desvelar o termo, recomenda-se a realização da análise de conceito, que é um “processo rigoroso de trazer clareza à definição dos conceitos usados na ciência”^(9)^. Esse método é amplamente utilizado na Enfermagem, com o propósito de subsidiar a pesquisa e a prática na área.

## OBJETIVO

Analisar o conceito de comportamento de risco para obesidade de acordo com o método de Walker e Avant.

## MÉTODOS

### Aspectos éticos

Este estudo não utilizou dados de domínio público nem envolveu seres humanos. Portanto, não houve necessidade de apreciação pelo Comitê de Ética em Pesquisa.

### Desenho do estudo

Trata-se de um estudo teórico, fundamentado no método de análise conceitual desenvolvido por Walker e Avant. Tal método é operacionalizado por meio de oito etapas, a saber: escolha do conceito a ser analisado, definição dos objetivos da análise, determinação das possíveis formas de uso do conceito, indicação dos atributos definidores do conceito, apresentação de um caso modelo, apresentação de outros tipos de casos, identificação dos antecedentes e consequentes referentes ao conceito e, por fim, designação de referenciais empíricos^(10)^. Salienta-se que foram realizadas as sete primeiras etapas, visto que estas foram suficientes para alcançar o objetivo do estudo.

O termo selecionado para análise foi “comportamento de risco para obesidade”. Essa escolha foi feita em decorrência da proximidade do autor com a temática obesidade e da necessidade de clarificar o referido conceito para avançar com o conhecimento e prosseguir com estudos nessa área. Foram definidos os seguintes objetivos para a análise:

- Desenvolver a definição de comportamentos de risco para obesidade;
- Determinar possíveis usos, atributos definidores, antecedentes e consequentes relativos ao comportamento de risco para obesidade.

Convém ressaltar que os antecedentes são considerados eventos anteriores ao conceito e precursores para o seu desencadeamento. Enquanto os consequentes são eventos posteriores ao conceito, que ocorrem em decorrência da presença dos atributos do conceito^(11)^.

Quanto à realização das demais etapas, estas foram pautadas em uma revisão da literatura, do tipo *scoping review*. Esta revisão seguiu as orientações do *JBI Manual for Evidence Synthesis* ^(12)^ e teve o protocolo registrado na plataforma Open Science Framework (OSF), disponível para consulta em https://osf.io/657j3/.

A priori, foram acessadas as plataformas *International Prospective Register of Systematic Reviews* (PROSPERO), Open Science Framework (OSF) e *The Cochrane Library*, com o propósito de identificar a existência de possíveis pesquisas cujo objetivo se assemelhasse ao da presente revisão. Contudo, não foram identificadas pesquisas congêneres.

### Critérios de inclusão e exclusão

Os critérios de elegibilidade incluíram estudos disponíveis na íntegra de forma gratuita, em qualquer ano ou idioma. Foram excluídos os estudos repetidos ou fora do escopo temático da revisão.

### Protocolo e período do estudo

Foram estabelecidas, com base na estratégia PCC, as seguintes questões de pesquisa: “Qual o conceito de comportamento de risco para obesidade? Quais seus usos, atributos, consequentes e antecedentes?”.

A seleção dos estudos para a elaboração desta análise conceitual foi realizada a partir do acesso ao Portal de Periódicos da Coordenação de Aperfeiçoamento de Pessoal de Nível Superior (CAPES), onde foram consultadas as seguintes fontes de dados: Biblioteca Virtual em Saúde (BVS), *Medical Literature Analysis and Retrieval System Online* (MEDLINE), Embase e Scopus. A busca foi realizada, de modo concomitante, por dois pesquisadores com formação em Enfermagem e especialistas em obesidade, ambos vinculados à Universidade Federal do Rio Grande do Norte (UFRN).

Para a conformação da estratégia de busca, foram utilizados os Descritores em Ciências da Saúde (DeCS) para a plataforma em português e os *Medical Subject Headings* (MeSH) para bases de língua inglesa, com exceção da Embase, onde foram utilizados os termos Emtree. Esses descritores foram associados aos operadores booleanos AND e OR.

Foram elaboradas, conforme as fontes de dados adotadas, as seguintes estratégias de busca:

- Busca 01 - realizada na BVS - (“obesidade”) OR (“Manutenção do Peso Corporal”) AND (“Comportamento de risco”);
- Busca 02 - realizada na BVS - (obesidade) AND (comportamento obesogênico);
- Busca 03 - realizada na Embase - *((obesity) OR (Body Weight Maintenance)) AND (‘high risk behavior’)*;
- Busca 04 - realizada na Embase - *((obesity) AND (“obesogenic behaviors”))*;
- Busca 05 - realizada na Medline e Scopus - *((obesity) OR (Body Weight Maintenance)) AND (Risk-Taking)*;
- Busca 06 - realizada na Medline e Scopus - *((obesity) AND (“obesogenic behaviors”))*.

Após a realização das buscas e a conferência dos resultados encontrados por cada pesquisador envolvido nesta etapa, as publicações foram exportadas para o software Rayyan (versão 2016, Qatar Foundation). Nele foi realizada uma triagem primária com base na leitura de títulos e resumos. Os estudos previamente selecionados foram lidos na íntegra para confirmar sua inclusão na amostra. A seleção foi realizada por um pesquisador enfermeiro especialista na área de obesidade.

### Análise dos resultados

A coleta de dados foi realizada por meio de um instrumento construído pelo autor, utilizando o programa Microsoft Office Word, versão 2016, no qual foram extraídas as seguintes informações: título, objetivo e tipo do estudo, ano de publicação, país, idioma, fonte de dados, usos, atributos definidores, antecedentes e consequentes relacionados ao conceito. Isso permitiu sumarizar os dados para caracterização dos estudos e construção dos resultados.

Os dados do estudo foram analisados sob o prisma da Análise de Conteúdo (AC) de Laurence Bardin. A operacionalização da AC constitui-se em três fases, a saber: pré-análise, exploração do material e interpretação dos resultados^(13)^. De modo sintético, a aplicação desta técnica ao contexto do estudo ocorreu da seguinte maneira: na primeira fase, o pesquisador realizou uma leitura preliminar dos estudos da amostra, na qual extraiu as primeiras impressões sobre o material e organizou mentalmente as informações acessadas. Na segunda fase, houve uma imersão nesse material e o desenvolvimento de processos específicos, como codificação e categorização das informações. Na terceira e última fase, o pesquisador realizou, com base na técnica de inferência, as interpretações do conteúdo, de modo a atribuir sentido às informações do estudo para a elaboração do conceito.

## RESULTADOS

Foram identificados 5218 estudos na busca inicial realizada somente com a inserção dos descritores e operadores booleanos, esse material foi exportado para o *software Rayyan* e em seguida foi empreendida a triagem dos estudos, em que houve a aplicação dos critérios de inclusão e exclusão, ao final foi obtida uma amostra de 16 estudos. O percurso de seleção está detalhado na Figura 1.

**Figura 1:** Fluxograma de seleção dos estudos para composição da amostra, Brasil, 2025

As 16 publicações selecionadas para a amostra final em sua maioria tratavam de estudos quantitativos do tipo transversal (50%) desenvolvidos em nove países distintos, dos quais se destacaram Estados Unidos da América (37,5%) e Brasil (18,7%). Os estudos foram publicados dentro do recorte temporal de 15 anos (2008 - 2023), com prevalência do ano de 2017 (25%) e tinham como público alvo adolescentes (75%), crianças (12,5%), adultos (6,25%) e trabalhadores (6,25%). Quanto ao idioma, os estudos estavam disponíveis em inglês (93,7%) e português (6,3%).

Referente a análise do conceito, verificou-se que o uso do termo comportamento de risco para obesidade foi maior na área da Saúde Pública (31,25%) e Nutrição (25%). Além disso, foram identificadas nomenclaturas alternativas como: fatores de risco comportamentais para o excesso de peso^(14)^, comportamento obesogênico^(15-17)^ e comportamento de risco para o excesso de peso^(18)^. Destaca-se que nenhum dos estudos apresentou definição de comportamento de risco para obesidade ou de qualquer termo equivalente.

Foram identificados 12 atributos, que estavam relacionados a três campos principais: alimentação, atividade física e hábitos gerais. Em cada campo, sobressaíram-se como atributos centrais, respectivamente, a ingestão de bebidas açucaradas junto a dietas ricas em carboidratos e alto teor de gordura (56,25%), a inatividade física (75%) e o uso excessivo de telas (56,25%). O Quadro 1 apresenta informações detalhadas sobre os resultados.

**Quadro 1:** Usos e atributos do conceito comportamento de risco para obesidade. Natal/RN, Brasil, 2025.

Usos do conceito
Saúde Pública^(14,15,18-20)^
Nutrição^(21-24)^
Medicina^(17,25,26)^
Educação Física^(27,28)^
Enfermagem^(16,29)^
**Atributos definidores do conceito**
**Relacionados a alimentação:** - Ingestão de bebidas açucaradas^(14,16,17,20-24,28)^ - Dietas ricas em carboidratos e alto teor de gordura^(14,19-25,28)^ - Baixo consumo de frutas e/ou vegetais^(16,17,20-26)^ - Não tomar café da manhã ^(14-16,18,20,24,25)^ - Consumo frequente de *fast food* e ultraprocessados em geral^(15,16,19-21,29)^
**Relacionados a atividade física:** - Inatividade física^(16-26,28)^ - Sedentarismo^(20,21,25,28)^ - Não participação das aulas de educação física^(24)^
**Relacionados aos hábitos gerais:** - Uso excessivo de telas^(14,15,17-21,26,29)^ - Tabagismo^(16,20,21,26,27)^ - Consumo de bebidas alcoólicas^(19-21,28,29)^ - Comer assistindo televisão ^(15,24,29)^

Após a seleção dos atributos foram construídos o caso modelo e o caso relacionado (Quadro 2). A elaboração destes casos é relevante para uma maior compreensão do leitor acerca do conceito, uma vez que, o caso modelo demonstra um exemplo prático de sua aplicação, enquanto o caso relacionado, ao passo que apresenta atributos semelhantes aos do conceito também possui elementos que não se adequam ao seu escopo quando analisados de forma aprofundada^(10)^.

**Quadro 2:** Caso modelo e caso limítrofe relacionados ao conceito de comportamento de risco para obesidade, Brasil, 2025

Caso modelo
A enfermeira Anna, ao realizar a coleta de dados antropométricos de escolares, atendeu A.S.M., adolescente de 16 anos, preto, com peso de 74,7 kg, altura de 162 cm, e escore Z-IMC de +2, o que o classificou com obesidade. Após identificar o quadro, a enfermeira solicitou ao adolescente uma conversa sobre sua situação de saúde. Durante a abordagem inicial, a profissional preencheu a ficha de consumo alimentar e discutiu os hábitos de vida, com o intuito de identificar comportamentos de risco para obesidade e, a partir disso, planejar uma intervenção. Durante a conversa, o adolescente relatou que não consumia frutas ou legumes, com exceção de batata, pois gostava muito e, inclusive, “comia bastante batata frita com cheddar e bacon”. Além disso, A.S.M. informou que preferia beber refrigerante ao invés de suco de fruta. Ele também mencionou não tomar o café da manhã, já que acordava sempre atrasado para a escola devido a noites mal dormidas. Ao longo do dia, o adolescente faz lanches rápidos, geralmente consumindo biscoitos recheados ou salgados fritos. No jantar, relatou que frequentemente se alimenta de macarrão instantâneo, por ser uma opção mais prática, uma vez que sua mãe trabalha fora e ele é responsável por preparar sua própria refeição. Por fim, o adolescente relatou que não pratica atividades físicas, nem mesmo durante as aulas de educação física na escola. Pelo contrário, ele prefere assistir séries ou jogar videogame.
**Caso relacionado**
V.C.O., 17 anos, sexo feminino, compareceu à Unidade Básica de Saúde para realizar uma consulta de planejamento familiar. A paciente é portadora da Síndrome do Ovário Policístico (SOP). Antes da consulta, foram aferidas suas medidas antropométricas, e verificou-se um escore Z-IMC de +3, indicativo de obesidade. A enfermeira responsável pelo atendimento iniciou a consulta abordando a situação nutricional da paciente. V.C.O. relatou estar ciente de sua condição e acredita que o excesso de peso tem relação com fatores genéticos, pois há frequência de casos de obesidade na família materna. Além disso, afirmou que seu quadro pode estar associado à SOP e/ou ao uso de antidepressivos. A paciente complementou o relato informando que não pratica atividade física e que passa muito tempo utilizando o celular. Diante do conhecimento prévio demonstrado pela paciente, foram realizadas breves recomendações sobre a importância da prática de atividade física e a redução do tempo de uso de telas. Ainda preocupada com o diagnóstico de obesidade, a enfermeira prosseguiu com a consulta, enfatizando o histórico familiar da paciente e as implicações da SOP.

Ressalta-se que dentre as possibilidades de caso complementar, o tipo relacionado foi escolhido tendo em vista que a literatura investigada apresentou inconsistência conceitual entre fatores de risco e comportamento de risco. Desse modo, o caso complementar ainda que apresente alguns elementos típicos do comportamento de risco para obesidade, a maioria deles tratam de fatores de risco, como idade, histórico familiar, doenças prévias e uso de determinados medicamentos. Para tornar mais evidente essa diferença foi elaborada a Figura 2, que demonstra a associação entre comportamentos de risco e fatores de risco para obesidade.

**Figura 2:** Sistema de fatores de risco para obesidade e sua associação com os comportamentos de risco para obesidade, Brasil, 2024

Em continuidade as etapas da análise do conceito, identificaram-se 11 antecedentes e seis consequentes. A influência do gênero e faixa etária (68,75%) e as condições socioeconômicas desfavoráveis (43,75%) foram os principais antecedentes, enquanto maior susceptibilidade ao sobrepeso/obesidade (100%) e aumento do risco de adoecimento por doenças crônicas endócrinas e cardiovasculares e cânceres (68,75%) destacaram-se dentre os consequentes. O Quadro 3 retrata na íntegra os dados encontrados.

**Quadro 3:** Antecedentes e consequentes do conceito de comportamento de risco para obesidade, Brasil, 2024

Antecedentes	Consequentes
- Influência do gênero e faixa etária^(16-18,20,22,24,27-29)^ - Condições socioeconômicas desfavoráveis ^(16-21,23,25,26)^ - Insegurança alimentar^(17,19,21,23,26)^ - Ambiente doméstico ou escolar obesogênico^(15,16,19,22,23)^ - Grupos de minoria sexual, racial ou étnica ^(14,17,26,29)^ - Falta de controle/monitoramento de responsáveis^*(14,15,29)^ - Ausência de apoio/estímulo para não realizar o comportamento^(19,20,21)^ - Necessidade de aprovação social^(16,19,27)^ - Influência da mídia social e propagandas de alimentos obesogênicos^(15,19,24)^ - Ambiente desfavorável à prática de exercícios físicos^**(17,19,21)^ - Pouco conhecimento nutricional por parte dos cuidadores^*(23)^	- Maior susceptibilidade ao sobrepeso e obesidade ^(14-29)^ - Aumento do risco de adoecimento por doenças crônicas endócrinas e cardiovasculares e cânceres^(15,17,19-22,24,25,28,29)^ - Danos à saúde física e mental nas fases posteriores do ciclo vital^(14-16,24,29)^ - Baixa duração e qualidade do sono^(15,16,18)^ - Morte prematura^(25,28)^ - Desencadeamento de outros comportamentos de risco^(20,24)^

Legenda:

* Específico para crianças e adolescentes /

** Foram consideradas as questões referentes a segurança do local e poucos espaços físicos.

## DISCUSSÃO

Como observado nos resultados, estudos sobre o comportamento de risco para obesidade têm sido realizados em diferentes partes do mundo, dado que o excesso de peso apresenta uma tendência global crescente, principalmente em países em desenvolvimento, como o Brasil^(30)^. No entanto, os Estados Unidos, o país que teve maior representatividade na amostra, apesar de não se enquadrar nesse perfil, ou seja, por não ser uma nação em desenvolvimento, também apresenta elevadas taxas de obesidade^(31)^.

Diante do agravamento dessa enfermidade e de suas repercussões na vida das pessoas e nos sistemas de saúde, pesquisas sobre a temática são cada vez mais necessárias. Todavia, observa-se uma prevalência de estudos de caráter quantitativo, como identificado na presente amostra, o que dificulta uma abordagem mais ampla dos aspectos subjetivos relacionados ao adoecimento, como, por exemplo, o comportamento^(32)^.

Há comprovação científica de que o comportamento adotado por cada indivíduo tem associação direta com a obesidade. Nesse contexto, torna-se claro que a investigação do comportamento de risco é um fator primordial para o enfrentamento da obesidade^(14-29)^. Apesar de sua relevância, o tema, no que diz respeito à sua perspectiva conceitual, é pouco explorado e, por vezes, confundido com outros conceitos.

Ainda no campo da saúde pública e da nutrição, onde o termo foi mais retratado, não há uma definição sólida. Os estudos analisados tendem a citar apenas exemplos de comportamentos de risco para obesidade, mas não abrangem uma definição precisa. A mesma situação ocorre em áreas como medicina, enfermagem e educação física, onde o uso dessa nomenclatura ou de sinônimos foi identificado.

A utilização do termo no campo da saúde pública reforça o reconhecimento de múltiplos fatores influentes na obesidade. Assim, a discussão nesse campo apresenta questões relacionadas ao desenvolvimento de políticas públicas com o intuito de enfrentar as situações que contribuem para aumentar o risco da obesidade, contrapondo-se, por exemplo, a empresas de qualquer setor que, por meio de seus produtos ou ações, favoreçam comportamentos não saudáveis^(33)^.

Já no campo das ciências da saúde (nutrição, medicina, enfermagem e educação física), é reforçada a imprescindibilidade do cuidado multiprofissional às pessoas com obesidade ou em risco de desenvolvê-la. Nesses casos, as recomendações do Ministério da Saúde indicam que os profissionais, principalmente aqueles atuantes na Atenção Primária à Saúde, como médicos e enfermeiros, estejam aptos a intervir no problema da obesidade e a identificar potenciais riscos que desencadeiam a doença, dentre eles o comportamento, não ficando essa responsabilidade apenas a cargo do nutricionista. Ademais, é estratégico incluir profissionais de educação física e psicólogos no enfrentamento desse problema^(8)^.

Em se tratando da perspectiva analítica do conceito, os atributos definidores do comportamento de risco para obesidade estão centrados na alimentação, na atividade física e nos hábitos gerais. A correlação desses atributos com a obesidade já era esperada e, inclusive, amplamente conhecida à luz da literatura científica.

Os estudos da amostra adicionam um novo conhecimento ao apontarem a necessidade de investigar esses comportamentos de forma integrada, e não de maneira dissociada. Considerando que a obesidade é uma doença multicausal, reforça-se a importância de analisar os comportamentos e, até mesmo, os fatores de risco em conjunto, para que as intervenções sejam mais eficazes^(16,20,21,24,28-29)^.

Os atributos definidores re lacionados à alimentação dizem respeito às práticas alimentares não saudáveis que contribuem para o adoecimento por obesidade. Essas práticas incluem a ausência de determinadas refeições durante o dia e a ingestão de alimentos com baixo teor nutricional, alto valor energético e elevada quantidade de açúcares e conservantes. Salienta-se que tais práticas geralmente diferem entre adultos, adolescentes e crianças.

Entre adultos, os comportamentos de risco para obesidade relacionados à alimentação estão associados a fatores como o preço dos alimentos, a falta de tempo para preparar refeições, a necessidade de motivação constante para aderir a uma dieta saudável e o baixo conhecimento nutricional^(34)^. Em adolescentes e crianças, os hábitos alimentares são influenciados por relações sociais no ambiente familiar e escolar, como a aceitação pelos pares e o controle dos pais. Este último fator é especialmente preponderante entre crianças, visto que as escolhas alimentares ficam a cargo de seus responsáveis ou cuidadores^(15,16,19,22,23)^.

Em relação aos atributos vinculados à atividade física, comuns a todas as faixas etárias, destacam-se o sedentarismo, caracterizado pelo baixo dispêndio energético em atividades como permanecer sentado ou deitado por grande parte do tempo diário, e a inatividade física, que corresponde à ausência de prática de atividades físicas^(35)^. Tais comportamentos podem ser revertidos por meio de diferentes estratégias, com destaque para as táticas de motivação^(36)^.

Outro atributo identificado, especificamente entre adolescentes, foi a não participação nas aulas práticas de educação física. De acordo com um estudo^(37)^, a não adesão a essas aulas é prejudicial, visto que os participantes das aulas de educação física escolar tendem a atingir maiores níveis de atividade física ao longo da semana em comparação àqueles que não frequentam essas atividades.

Por fim, a última categoria de atributos faz menção aos hábitos gerais relacionados ao comportamento de risco para obesidade, como o uso de telas, o tabagismo e o consumo de álcool. Segundo autores^(20)^, embora a literatura enfatize os comportamentos alimentares e sedentários, o uso de telas e de substâncias deve ser considerado um fator fundamental durante a abordagem de pacientes com obesidade ou em risco de desenvolvê-la.

A exposição demasiada a telas é um dos comportamentos que merece maior destaque, pois desencadeia outros comportamentos de risco. Esse hábito é apontado como um marcador para o sedentarismo, ocorrendo com maior frequência entre o público infantojuvenil e contribuindo para quadros de obesidade e sobrepeso^(17,29)^.

No que se refere aos antecedentes do conceito, a maioria foi relacionada a aspectos de caráter demográfico, nutricional e socioambiental, destacando-se questões como gênero, idade, etnia, condições socioeconômicas, insegurança alimentar e ambientes obesogênicos. Outros antecedentes, que não se adequavam a nenhum desses grupos, estiveram presentes em menor quantidade na amostra.

Não houve consenso entre os estudos que analisaram a influência do gênero sobre os comportamentos de risco; entretanto, uma parte considerável afirmou que pessoas do sexo masculino estão mais propensas a desenvolver esses comportamentos^(16,20-22)^. De forma associada, os adolescentes foram identificados como a faixa etária mais suscetível a adotar comportamentos de risco à saúde de modo geral, muitos dos quais são considerados preditores de obesidade^(15-21,26,27,29)^.

Quanto à insegurança alimentar, esta ocorre quando os princípios da segurança alimentar são comprometidos, ou seja, quando não são asseguradas condições adequadas de acesso, qualidade e quantidade de alimentos. Vale ressaltar que existe uma relação entre condições socioeconômicas precárias e insegurança alimentar, sendo ambas potenciais fatores para o desenvolvimento da obesidade^(38)^.

Em relação ao ambiente obesogênico, este pode ser caracterizado como um ambiente que influencia negativamente o indivíduo, incentivando hábitos alimentares inadequados e comportamentos sedentários, o que culmina em prejuízos à saúde^(39)^. Assim, não há um espaço exclusivo que se caracterize como obesogênico; qualquer local que se enquadre nessa definição pode ser considerado como tal.

No âmbito escolar, estudos indicam que a presença de locais que ofertam alimentos ultraprocessados e *fast foods* dentro ou ao redor das instituições, aliada à infraestrutura precária, marcada pela falta de espaços adequados para a realização de atividades físicas e pela insegurança do local, são fatores apontados como principais responsáveis pela adesão a comportamentos de risco para obesidade entre escolares^(40)^.

Adicionalmente, destacam-se situações relacionadas à influência de amigos e à necessidade de aceitação pelos pares, que levam adolescentes a adotar práticas alimentares inadequadas e outros tipos de comportamentos de risco como meio de alcançar validação social^(41)^. Soma-se a isso a atuação da mídia, que promove propagandas e conteúdos que normalizam hábitos alimentares inadequados, tornando-os corriqueiros^(42)^.

Diante desse cenário, os pais podem contribuir de maneira positiva ao estimular crianças e adolescentes no desenvolvimento de hábitos saudáveis, como a construção de uma alimentação equilibrada, a prática regular de atividades físicas e o monitoramento do tempo de uso das telas. Ademais, os pais são exemplos importantes para seus filhos e, por isso, também devem adotar condutas saudáveis^(43)^.

Acerca dos consequentes do conceito, é fundamental ressaltar que sua identificação fornece uma visão abrangente das complicações e agravos relacionados à obesidade, os quais são fortemente influenciados pelos comportamentos de risco. Esses consequentes evidenciam a propensão dos indivíduos que adotam tais comportamentos ao desenvolvimento da doença e aos danos físicos e psicológicos associados, culminando em uma baixa qualidade de vida. Esses efeitos podem se perpetuar na vida adulta caso não haja intervenções adequadas.

Diante dessa situação, um estudo^(44)^ reforça que as intervenções devem ser realizadas o mais precocemente possível para evitar o desenvolvimento de complicações. Assim, a prevenção primária, especialmente por meio de intervenções educativas, é uma estratégia pertinente para alcançar bons resultados no enfrentamento do problema, como demonstrado em outra pesquisa^(45)^.

### Limitações do estudo

A principal limitação deste estudo foi o uso exclusivo de fontes eletrônicas para a busca dos estudos, associado à ausência de consulta à literatura cinzenta e à exclusão de muitos trabalhos devido à falta de acesso gratuito, mesmo com a execução das buscas ocorrendo por meio do Portal de Periódicos da CAPES. Ademais, destaca-se a dificuldade em encontrar descritores e associá-los de modo que refletissem adequadamente o tema em questão.

### Contribuições para a área da Enfermagem, Saúde ou Política Pública

A análise conceitual do termo *comportamento de risco para obesidade* fornece subsídios teóricos para apoiar o pensamento crítico, a prática baseada em evidências e o raciocínio diagnóstico na assistência de Enfermagem. Além disso, favorece o entendimento dos fatores subjacentes à obesidade, permitindo ao enfermeiro atuar como um agente promotor da saúde e direcionador de um estilo de vida saudável em quaisquer serviços da Rede de Atenção à Saúde.

## CONSIDERAÇÕES FINAIS

A presente análise, realizada à luz do método de Walker e Avant, possibilitou a clarificação do conceito de *comportamento de risco para obesidade*, que até o momento permanecia indefinido ou, pelo menos, subentendido. Com base na literatura consultada, os autores definem *comportamento de risco para obesidade* como a ação desenvolvida pelo indivíduo, que ocorre com maior frequência na adolescência, mas não exclusivamente nessa fase, tornando-o mais suscetível à obesidade e suas complicações, como o adoecimento por doenças crônicas cardiometabólicas e maior risco de morte precoce.

Ao buscar a definição deste termo, foram identificados seus antecedentes, atributos definidores e consequentes, proporcionando uma imersão profunda no conceito e um conhecimento mais abrangente. Isso permitiu aos autores romper com associações inadequadas ao conceito, que eram frequentemente empregadas de maneira imprecisa.

Ademais, sugere-se que pesquisas futuras validem o *comportamento de risco para obesidade* como um diagnóstico de Enfermagem, considerando que taxonomias que subsidiam a prática teórico-clínica do enfermeiro, como a *North American Nursing Diagnosis Association* (NANDA), já apresentam diagnósticos relativos à obesidade.
